# ‘I couldn’t say the words’: communicative bodies and spaces in parents’ encounters with nonsuicidal self-injury

**DOI:** 10.1057/s41285-020-00144-y

**Published:** 2020-06-17

**Authors:** Peter Steggals, Steph Lawler, Ruth Graham

**Affiliations:** 1 Newcastle University, Newcastle upon Tyne, UK; 2 University of York, York, UK

**Keywords:** Nonsuicidal self-injury, Self-harm, Social interaction, Intercorporeality, Phenomenological sociology

## Abstract

There is a growing recognition that nonsuicidal self-injury commonly incorporates communicative and interactional dimensions. But regardless of whether we approach self-injury within the terms of deliberate interpersonal communication, it is undeniably something that conveys a significant impact into the social and communicative field between people. As such, it is something that can be approached and analysed as communicative in this more general sense. In this paper, we draw on 13 in-depth qualitative interviews with the parents of people who self-injure, conducted for a larger pilot study, to explore some of these more general communicative processes, spaces and impacts associated with self-injury. By providing a phenomenologically informed examination of parents’ experiences, we argue that self-injury is in fact a richly communicative phenomenon, albeit one that cannot be adequately mapped using the traditional sender-receiver communication paradigm. To provide a more nuanced mapping, we look beyond this paradigm to include more subtle, ambiguous, pre-reflexive and bodily forms of communication. Indeed, self-injury offers a particularly powerful case study with which to think through a more complex model of communication, one that connects the interpersonal, intersubjective and intercorporeal levels, and that, as such, is more appropriate to the sociologies of everyday life and embodiment.

[B]odily behaviour is meaningful, it is intentional, and as such it is neither internal nor external, but rather beyond this artificial distinction - Dan Zahavi (2001, p. 153)

## Introduction

Research on nonsuicidal self-injury tends to be dominated by an almost exclusive focus on the *individuality* of the person who self-injures. Where social context is considered, it is usually cast as a set of secondary biographical correlates to the primary inner facts of the matter; with the figure of the individual taken as both the sole cause and exclusive site of the self-injury (Chandler 2016; Steggals 2015). So embedded is this individualistic framing, that examples of self-injury perceived to be insufficiently private, and therefore individual, are commonly dismissed as inauthentic and ‘attention-seeking’ (Chandler 2016; Scourfield et al. 2011; Steggals et al. 2020). However, while a focus on the inner condition of the person is clearly important (Klonsky 2006), the *exclusivity* of this focus is both historically contingent (Millard 2013) and empirically questionable (Brossard 2018; Chandler 2016; Steggals 2015). One potential challenge to this exclusivity has come from research that suggests self-injury may contain interpersonal as well as intrapsychic elements (Brown et al. 2002; Nock 2008; Nock and Prinstein 2004, 2005; Rodham et al. 2004; Turner et al. 2012). We have ourselves recently written on this (Steggals et al. 2020), and have argued that self-injury *should* be thought of as both a personal *and* a social practice; one that often incorporates an important relational, interactional or—otherwise put—a *communicative* dimension. But regardless of whether we should approach self-injury (at least partially) in terms of interpersonal communication, it is undeniably something that significantly impacts on the social and communicative field that exists between people (Noch, 2008; Steggals et al. 2020; Turner et al. 2012). As such, self-injury is something that *can be approached and analysed as communicative in this more general sense.* And it is toward this more general sense that we turn in this article.

Of course, what we mean by ‘communication’ is of central importance here. In the relevant academic literatures, communication has consistently been a contested concept (Dance 1970; MacKay 1972). However, within this contested theoretical space, a strongly influential tradition, paradigmatically associated with Shannon and Weaver’s highly linear model (1949), restricts communication to only one part of the full range of informative, expressive and indexical performances that people generate. Within this paradigm communication is a conscious and deliberate act which transmits representational information from one person (the sender) to another (the receiver). According to this paradigm, self-injury is not communicative, unless it is performed with the active and conscious intention to transmit a particular message to someone capable of decoding it—precisely the idea of self-injury that is commonly put in question by terms like ‘inauthentic’ and ‘attention-seeking’.

However, much recent work in communication studies has problematised this linear, sender–receiver paradigm (Meyer et al. 2017). The notion of a monadic, bounded and utterly private individual that acts as a ‘signalling device by which cognitive states, intentions, and mental imagery are expressed’ has been challenged. In its place we find a more ‘holistic or multimodal’ appreciation of how ‘an interacting human body resonates, entrains, or even merges with another’ (Meyer et al. 2017, p. xv). This latter perspective, influenced by both Merleau-Ponty’s phenomenology (1962, 1968, 1971) and Goffman’s interactional sociology (1963, 1967) pictures a kind of shared co-embodied system (Bateson 1972): a matrix of communication and coordination within which everyday life is pursued and interactions facilitated, but which nonetheless remains largely unnoticed in the taken-for-granted background of conscious awareness. According to this alternative paradigm, though we may not realise it, ‘we cannot stop communicating’ (Goffman 1963, p. 35) and so the idea of self-injury as a completely individual, secret and private practice that lacks any communicative dimension, may be as fundamentally unworkable as the idea of a strictly private language (Wittgenstein 2009 [1958]).

In this article, we explore some of the communicative processes, spaces and impacts associated with self-injury, utilising a phenomenologically informed examination of parents’ experiences of living and interacting with children who self-injure. We argue that, beneath the level of interpersonal communication the social life of self-injury is rich with complex, subtle but often ambiguous communicative activity. In order to understand this activity, we must move beyond our attachment to the *sender–receiver* paradigm and work with the newer multimodal approach. We must also move beyond the associated idea of the bounded and monadic individual, or what Elias famously called the *homo clausus* (Elias 1994 [1939]): replacing its familiar dichotomies of the inside/outside and the private/public with a more fluid model of the always already intersubjective; and replacing its solid boundaries with a more porous membrane of active, if not always fully conscious, communication. In its place then, we propose something more like what O’Neill (1989) and Frank (1991) have called ‘the communicative body’. But it is important to note that approaching self-injury in this way does not mean denying that it is typically a deeply personal or even secret matter. Rather, it means acknowledging that neither our common ideas nor our scientific understanding of private life and personal secrets implies an absolute and hermetically sealed individualism (Manen and Levering 1996).

## Methodology

The research reported here emerged from a 2016-2017 English pilot study that explored the degree to which significant relationships shape people’s practices and experiences of self-injury, and how these relationships are themselves affected and shaped by self-injury. Twenty-six in-depth qualitative interviews (20 with women and 6 with men) were conducted with both people who self-injure (n = 9), people who are in relationships with people who self-injure (n = 12), and people who have had both experiences (n = 6). The overall aim was to better understand and describe the interaction between self-injury and the key relationships forming its immediate social context. We interviewed nine mothers and three fathers, ranging from 43 to 75 in age. Ten of these parents had not themselves self-injured, while two of them had their own history of self-injury. The analysis presented here represents one key strand of the findings from the overall study.

Our analysis of the communicative life of self-injury is structured by reference to three levels of possible communication: the *interpersonal*, the *intersubjective* ^1^ and the *intercorporeal*. These three conceptual lenses are used to disclose and analyse the communicative dimension present within our participants’ accounts, as well as to understand how reflexively aware they were of such communicative processes and their effects. Though, as already mentioned, we have put the question of whether self-injury can ever be thought of as an unambiguously deliberate interpersonal communication to one side for the purposes of this article, it is necessary nonetheless to look at what is going on at the level of *interpersonal communication* where self-injury can be correlated with changing patterns of normative and linguistic interaction. *Intersubjective communication* describes the range of reflexive and expressive practices that facilitate interpersonal communication, but which are characterised by an ambiguity in communicative intent. And *intercorporeal communication* describes multiple pre-reflexive processes that allow one body to resonate with another, creating the communicative context of a visceral ‘we’ that enables more explicit and reflexive forms of communication to occur (Meyer et al. 2017; Schutz and Luckmann 1973). Following Merleau-Ponty then, we treat *intercorporeality* as a more primary and basic form of inter-bodily connection than *intersubjectivity,* which he associated with the imagined construction of the other within the ego structure of the self (see for example, 1968, p. 180;also Loenhoff 2017). Taken together, these three levels of communication comprise a holistic approach that necessitates a move away from the sender—receiver communication paradigm, along with the idea of the *homo clausus* that underpins it. Below we apply each level of our analytic model to our empirical data, and ultimately demonstrate our argument that while self-injury may be experienced as intensely personal, it must nonetheless be understood as something that is also richly communicative.

## Interpersonal communication

self-injury has often been connected by researchers and clinicians to a breakdown in interpersonal communication, the ‘failed promise’ (Kilby 2001) of normative language to support social recognition (Frank 1991). Nock, for example, has argued that in some cases self-injury may function as a kind of ‘high intensity social signal’ used to communicate in circumstances where other communication strategies such as talking, yelling, and crying are perceived to have failed (2008, p. 159). self-injury can ‘voice things that cannot be said’ (Pembroke 1996, p. 45), becoming what Hewitt calls ‘the language of blood and pain’ (Hewitt 1997, p. 58).

Those beginning a period of self-injury often withdraw from others and cease their normal patterns of communication, which typically represents the first impact of self-injury on the communicative field of the family. The parents who contributed to this study routinely reported such a withdrawal, occurring even before the selfinjury became known. Usually, this was interpreted as an expected part of parenting a teenager. Rabindra for example reports that his teenage son, Carl, became less communicative but noted ‘you put that down to er, growing up, because you start developing your own interests and hanging out with your friends and whatever. So, I didn’t sort of think there was anything unusual’. Unusual or not, this experience can be a traumatic transformation for parents. Paula noticed a change in her daughter Mary at around 14 years of age: ‘it was like one moment I had this little girl and the next moment I had this… stranger… This really angry stranger. And like, what happened? What, what happened in such a short space of time? And, and it was almost like you grieved’.

Rachel, a 47-year-old, single mother of two daughters, struggled with changes in her youngest daughter’s behaviour when she, Mia, became more withdrawn and less communicative at age 13 or 14. Rachel also understood this as a natural part of the growing process, a manifestation of teenage angst and the desire to renegotiate the child-parent relationship, but eventually discovered that this social withdrawal helped Mia hide her depression, suicidal feelings and self-injury. After Mia’s self-injury has been discovered, the breakdown in normative language spread from daughter to mother. As Rachel explains: ‘I couldn’t really say the words… I couldn’t use the word ‘self-harm’, I couldn’t, or ‘hurt yourself’, or ‘cut yourself’; I couldn’t use those words, they couldn’t come out of my mouth, they were too painful’. Language had become highly sensitised, charged and even dangerous.

Other parents reported similar concerns about the use of words in the context of self-injury. Barbara, a 43-year-old mother of a teenager who had been self-injuring, noted ‘I just felt like anything I said would be the wrong thing to say’. Rabindra and his wife, Ingrid, who were interviewed separately, echoed this sentiment. Ingrid explained that when she found out about Carl’s self-injury ‘it left me speechless’. For the eleven years since, during which Carl continued to self-injure, she described her experience as.

walking on eggshells because you don’t know what you can say to him or not, because you don’t know whether it will set something off so you don’t know whether you would say to, you could say to him the same things you could say to the other two [of her children] without there being a backlash of some form or other, because he won’t necessarily argue it out with you, and I find that quite frustrating … with Carl you always feel like you’ve got to approach it from a slightly sideways angle and sort of, you know, it’s like, it’s almost like you need to put him in a sort of safe place in a padded cell first so that, you know, he feels secure and you sort of come out with 35% of what you want to say, hope that he reads a little bit between the lines and that 50% of what you want to say actually gets delivered

The failure of, and sensitisation to, normative language, together with the associated social withdrawal, represent a partial collapse of interpersonal communication within a particular relationship network—such as the family home. Such disturbances in the communicative field are inevitably communicative in themselves. Rabindra for example explains.

we now know conditions, circumstances, patterns of behaviour that we think we know that something is coming … We’re quite sort of, your nose gets attuned to it … We can pick it up in different ways um, we all, usually if we’re down in the country [at their second home while Carl remains at their first] we’ll speak at least once a day type of thing, once every couple of days, and if we haven’t heard from him for a week, or we try to call him and he hasn’t called back you know something’s up.

In this way, something as thoroughly uncommunicative as a resolutely closed door, which appears to be a *refusal* to communicate, becomes a communication in itself and takes on great significance—a drop in mood indicating a rise in danger. As Ingrid explains:

he’s got to the point where when things are really bad he’ll lock himself in his room for several days, you just don’t really see him … He’ll just disappear, you know he’ll come and get some drink and some food when you’re not in the room/the flat. Um, and you know he’s been around, he’s, he’s, you know, he’s alive, he’s out there, he’s up there but you won’t see him in the daytime, he won’t sit for a meal or, you know, he’s not hungry at that point, you know. You don’t know when he wakes up either, so he hides away. You know, it could be two or three days.

## Intersubjective communication

Writing on self-injury, Kilby argues that, ‘if the promise of language fails and speaking cannot sustain life, another ‘voice’ must be found, especially when faced with the need to testify to the traumatic conditions of life itself’ (2001, p. 125). This is the realm of what O’Neill (1989) and Frank (1991), writing more generally, call the ‘communicative body’. When normative language can no longer support our need for social recognition, the body ‘breaks out of [the] codes’ that have silenced the subject and seeks ‘self-expression in a code of its own invention’ (Frank 1991, p. 85). self-injury could itself be (partially) understood in this way (Kilby 2001; Nock 2008), as a kind of ‘bright red scream’ (Strong 2005). But the general pattern of everyday life is saturated in various signs, signals, and codes and these do not cease just because normative interpersonal communication has failed, and someone has begun to self-injure. Non-interpersonal activities like body-language fill the communicative gaps left by the failure of normative language, supporting the bodily code and carrying its non-normative themes and content. Indeed, the power of self-injury to function as a bodily code may itself have an amplifying effect on the communicative power of non-interpersonal communications. And it is not just bodies which are communicative, but spaces also, such as the resolutely closed bedroom door noted above. A more developed, and possibly more deliberately communicative, example is described by Rachel:

I used to go into her bedroom and I’d, I’d look. I used to have a Monday off back then, and when she was at school on a Monday I used to go up to her bedroom and just look to see if I could find anything she’d be hurting herself with in there … She used to keep them um, tiny little plastic bags with them in and she’d, she’d just hide them. And occasionally I’d find them like, Sellotaped-up to the top of the door frame and things like that … I’d asked her about them. I’d say: ‘oh I noticed, you know, you’ve got some blades in such and such a place’. But I never took them.

Crucially, while Rachel would not remove the razor blades she *would* let Mia know that she had found them. So, while these items were hidden from sight, Mia knew her mother was going into her room *and nonetheless continued to leave items there to be found.* This suggests a communicative process at work, where Mia’s bedroom works as an alternative bulletin board in which things left, and things discovered enabled an alternative mode of indirect communication. Indeed, Mia even told her Childhood and Adolescent Mental Health Services (CAMHS) worker to tell her mother where, in Mia’s bedroom, she could find some old suicide notes that she had written, something she could not talk to her mother about directly.

Paula described a similar situation with her daughter, Mary:

she started shutting herself away in her bedroom. Um, I’d clear the bedroom, I’d find the tissues um, with blood, and that used to bother me, used to make me feel sick. And then I found um, there was like letters and things where she kind of left them out, I think, for me to see … I was always looking for signs.

While Mia’s and Mary’s bedrooms *did in fact* become a kind of communicative zone, how intentional this communication was is ambiguous, and in turn, ambiguity is characteristic of intersubjective communication within the context of self-injury. For example, the moment that self-injury is disclosed to another is often marked by ambiguity. Rachel discovered Mia’s self-injury when Mia put on a ‘strappy little top’ and went sunbathing in the garden, revealing her arms and her scars:

I remember saying to her, my mum was outside, and I obviously didn’t want to draw attention, so I just said to her: ‘oh, can I, can we just go and have a chat inside in a minute?’ She knew I’d clocked it, she’d know, but she’d obviously, she’d put a strappy top on, so she must have known I was going to see that day … Um, so you know, whether that, that element was, you know, she was ready to, to share that information with me I guess.

Rachel clearly interprets Mia’s actions as deliberately communicative, but this is an interpretation and the ambiguity is obvious. Likewise, as Ingrid recalls.

the fact that [Carl] self-harmed only came to light … [b]ecause they were re-vamping the common room er, painting it, he happened to wear some short-sleeved t-shirts which then showed some scarring, at which point the housemaster rang us and said, you know ‘were you aware?’ And we certainly weren’t.

[Steggals]: Yeah. So, there must have been a period where he was doing this very privately and nobody knew.

Ingrid: Yeah, he always has done to be honest. This is the first year since maybe six months ago where he doesn’t mind wandering around anywhere, except in front of his grandmother, with short sleeves on because the scarring on his left arm was quite substantial and some of it’s quite, quite deep and, um, but until then he always hid it and, you know, looking back at that time when we got the phone call, he’d have the occasional sort of sweatband that he’d wear. Um, seemed a bit weird but, you know, kids do all sorts of things. But when we look back, where he wore the sweatband you can now see that there’s scarring underneath, so he hid it and he wouldn’t wander around in a short-sleeved t-shirt and we’d say ‘good grief, it’s 25 degrees out there, what are you doing? You know, normally you’d wear shorts and a t-shirt, how come you’re wearing a shirt?’ And then you can add it all up afterwards. At the time: no idea.

While Carl’s wearing a t-shirt that day could have been a mistake, it does not fit the overall pattern of attentively hiding his scars, and so seems suspiciously like an intersubjective, albeit ambiguous, communication. Indeed, such ambiguity is quite characteristic in people’s reflections on the communicative status of self-injury. And this remains the case even when it is the person who self-injured who is reflecting on their own past actions. Perhaps ambiguity is not just a problem that clouds issues of intent and interpretation then, but an element of how such patterns of behaviour work *as forms of communicative action*, short-circuiting the weight and implications of more normative, obviously deliberate communication.

## Intercorporeal communication

While we can recognise the ambiguity of both Mia’s actions when she wore the ‘strappy top’, and Rachel’s interpretation of them, Rachel nevertheless does seem to feel that Mia’s actions were in some sense deliberate and that she had decided to share the secret of her self-injury. However, Rachel was not ready to share the secret with her own mother, Mia’s grandmother, and described controlling her own reaction to the discovery of self-injury so as to not ‘draw [her mother’s] attention’. In fact, Rachel successfully kept Mia’s self-injury a secret, not just from Mia’s grandmother, but also from Mia’s father and Mia’s sister. Rachel didn’t see Mia’s disclosure of self-injury as inauthentic, as now being *shown* rather than *secret*, or *public* rather than *private.* Rather, she re-drew the boundary of the secret and the private so that they stopped being wholly individual (to Mia) and now included herself as well.

So, while we may think of secrecy in self-injury as something being kept hermetically sealed within the boundaries of the individual, our data suggest that a secret shared remains a secret nonetheless; carrying the same power to mark off social space as before. What changes is the division of who is marked off as *inside* the secret, and who *outside.* Rachel became involved in some of the same practices and performances of secrecy that Mia has previously been pursuing alone, albeit filtered and articulated through the social identity and concerns of the parent. And this includes the anxiety of discovery and sense of shame that comes with a secret stigma. Similarly, Barbara for example,told Steggals about how her daughter, Monica, was ready to drop the secret and consider her scars as part of her public identity, but for Barbara the scars were a source of embarrassment and the judgmental gaze of outsiders:

Almost like she [daughter] had gone through it and she had dealt with it, and this is what she’s left with, and she’s not going to hide it and be ashamed of herself any more.And that’s how she felt, and I felt differently; I was thinking ‘just cover them up’ … Because I don’t want to have to deal with people’s looks of her being judged and then me being judged. Because people do, they think ‘oh, they obviously haven’t been very good parents then, if they’ve done that. They haven’t kept them safe’ or ‘what sort of home life have they got if the children are doing that to themselves?’ And people do judge, and they make wrong judgements and assumptions.

Such anxiety involves complexity, as Donna (a woman in her early 60s with a teenage daughter) notes in her account of the ‘the burden of secrecy’:

There are a number of things going on. I have to respect Karen’s [daughter’s] right to privacy. But I so want to talk to somebody. I want to share and maybe get absolution from my listener who will tell me that I’m not a bad mother after all. And then again - maybe I’d rather do without the absolution and just keep the secret so that nobody else is tipped off to what a failure I’ve been. So, I want to keep the secret and I want to share it too.

This anxiety about being blamed is of course an entirely rational one, since parents, especially mothers, are generally held to be responsible for bad things in a child’s life (though rarely credited for good ones: Lawler 2000). The well-being of the child, it is often assumed, is entirely within the gift of the parents, and especially the mother. As Ann-Marie Ambert has argued:

[W]hen one sees children, one ’sees’ parents. When one sees children who have problems, one looks for parents, especially mothers. When one seeks solutions to children’s problems … one immediately turns to parents who are then scrutinized by a variety of establishments (Ambert 1994, p. 530)

The mothers in our study can hardly be unaware of this, and Donna’s wish for ‘absolution’ is entirely understandable, as is her contrary wish to keep her ‘failure’ secret. And her daughter, similarly, is likely to be aware of the patterns of knowledge and scrutiny in which both mother and daughter are bound up. This can mean a complex relationship with the health professionals who entered several participants’ lives: welcomed as a source of help, their presence may equally have brought the most profound anxieties about being ‘blamed’. Paula’s discussion of her daughter Mary’s experiences with a CAMHS worker speaks to some of these issues:

she got really cross with him [CAMHS worker] because he wasn’t focused on that [points to forearm] he was more focused on her relationship with me and her dad … And the whole session and at the end of it she said ‘look,can we leave my parents out of this? They’re great. This isn’t about them, this is about me and I’ve got other issues going on at school, and boys and whatever’ and she didn’t go back after that. She said it was like he was always trying to put words in my mouth. She said ‘I didn’t like it’. He was searching for something that wasn’t there.

This looks at first sight like self-justification, and possibly it is. But Paula was highly reflexive about her mothering and did in fact blame herself (she said for example that she had altered her mode of relating to Mary). In addition,parental influence is unlikely to be the only factor in a young person’s desire to self-injure. Furthermore, alongside secrecy and shame, parents typically experienced anxiety and the sense of a familiar world becoming strange and threatening.Once she discovered Mia’s selfinjury, Rachel explained that:

I used to go round my house and look at every potential danger. She could burn herself on a light bulb, she could do anything. I could not take, I couldn’t safeguard her, and at that, I used to get so frustrated about that because I used to literally walk round my house and look at everything and just think ‘well, she could hurt herself on that’ … there’s no way you could remove everything that she could harm herself with. You know, you’d lock away the paracetamol and the drugs and you’d, you know, there was a point where I kind of, very sharp knives, they, they went out the house. I locked them in the garage, you know … And I just thought: ‘why am I doing this? Because, actually, there’s toilet cleaner there, there’s bleach; I cannot remove everything from this house that she could, you know, either hurt herself with or poison herself with or, you know, I can’t do it.

At a more general level, Rabindra described the ‘nose’ that he and Ingrid developed, a kind of permanent vigilance, tuned in to the communicative field of the household, waiting for signs of a coming episode of self-injury. Experiences of anxiety and shame, and the practices and concerns of secrecy, may not seem overtly communicative. But when taken with the kind of anxiety, estrangement from the familiar, and constant vigilance that we discussed previously, they represent an interesting case of communication, in the way that a disease may be *communicated* or passed on, a vector for negative affect. These feelings, concerns, dilemmas and sensitivities are, while filtered and mediated by the parental role, nonetheless the same feelings and sensitivities that are traditionally associated with someone who is self-injuring. This kind of communication then runs well below the traditional sender–receiver model and suggests a kind of *intercorporeality* or inter-bodily resonance, an ‘entanglement between self and other’ (Dolezal 2017, p. 320). There is a sense in which these difficult feelings and lived experiences had been communicated from child to parent who has taken them on, albeit from her own position and point of view. What might conventionally be considered ‘private’, without becoming public, becomes a shared state and experience.

## Toward a (co-)embodied phenomenology of self-injury

In Saussure’s classic *Course in General Linguistics,* there is a famous diagram of two disembodied heads with the brain and mouth of each connected by wire-like lines to the ears of the other (2013 [1916], p. 14). In many ways the diagram nicely illustrates the concept of the *homo clausus*: both individuals are closed and sealed into their own inner worlds; information about thoughts, moods and feelings are inaccessibly locked up inside the private inner space of each person, unless and until that person chooses to communicate this information to another. But obviously the diagram also speaks to the linear sender–receiver communication paradigm that we mentioned in the introduction. Indeed, the wires in this diagram recall that Shannon and Weaver’s highly influential version of this paradigm grew out of their wartime experience in the Bell Telephone Laboratories (Shannon and Weaver 1949). And if we understand communication in this telephonic way, then we must think of self-injury as something that is not communicative at all: a completely closed psycho-affective and behavioural circuit. Of course, this is not an uncommon model, in either public or professional discourse about self-injury (Chandler 2016; Steggals 2015; Steggals et al. 2020), but it is wholly inadequate for understanding the subtle and ambiguous excitations in the communicative field of the family hone that we have described above.

The key to this apparent paradox is that both the *homo clausus* and the sender–receiver communication paradigm are not just abstract ideas, but potent discourses that inform and regulate the normative conditions of people’s experience, as well as the psychosocial patterns of their subjectivity (Foucault 1991 [1975]). This is why we find these discourses evident in the thoughts, feelings and practices common to self-injury. And, if we follow Steggals’ argument (2015), it is the reason why self-injury can even be understood as a kind of symbolic crystallisation or hyperbolic performance of precisely these discourses. But, even if our personal and cultural imaginary is formed through such individualistic discourses, we must remember the inherent irony that these are nonetheless *social discourses*: they shape and regulate the pattern of our subjectivity, but they do not determine its formal structure (see for example Turner’s ‘ontological foundationalism’, 1992; also O’Neill 1989). So, as Siri Hustvedt points out, no matter how we may imagine ourselves, we ‘are not closed, wholly autonomous creatures. We are born of another person and open to others from birth on, this openness is by its very nature ambiguous, reciprocal, and mixed’ (2017, pp. 380–381). This ofcourse is a longstanding perspective in sociology, axiomatic in thework of Simmel (2009 [1908]), Elias (1994 [1939]) and Goffman (1963, 1967, 1968). But it is also key to the phenomenology of Merleau-Ponty (1962, 1968) who makes clear that this openness to others has major implications for how we think about the communicative power of social life under conditions of self-injury: he explains that ‘[w]e must reject the prejudice which makes inner realities out of love, hate or anger, leaving them accessible to one single witness; the person who feels them. Anger, shame, hate and love… exist on this face or in those gestures, not hidden behind them’ (1971, p. 52).

This is the picture we believe our data demonstrates: even as our participant’s stories describe people closing down at the level of normative interpersonal communication, of the wires between sender and receiver being disconnected, what we find is not the erasure of communication but rather the *disclosure* of the many ambiguous, reciprocal and mixed communications that exist beneath this level and that are articulated through a fundamentally communicative body. To understand how this works between people and within spaces affected by self-injury, we need to move beyond the *homo clausus* and the sender–receiver communication paradigm: in fact, we must complete Saussure’s diagram ‘by drawing out the moving bodies underneath the talking heads’ (Meyer et al. 2017, p. xvi).

This of course, is a significant and ongoing challenge. In our remaining space we restrict ourselves to noting four key elements of this challenge that help to interpret our data picture and position it within a broader intellectual context. These are: that the body is a foundational part of our subjectivity; that the body is extended in living space; that the body is essentially relational; and that the body is communicative.

### The body as foundational

Drawing the bodies out on to Saussure’s diagram should not be done by simply adding them on to a pre-existing and atomic self but rather by disclosing what was there all along: that the body is a not just a fundamental but also a *foundational* part of our subjectivity. This of course has been a key insight in embodied sociology (Williams and Bendelow 1998; see Crossley 2001; Turner 2008), phenomenology (Merleau-Ponty 1962, 1968, 1971; Gallagher 2005), and in research on intercorporeality (Meyer et al. 2017). Bodies are not simply vehicles or signalling devices under the control of an autonomous and interior mind, mere objects or *ȁKorper* to use Husserl’s term (2002 [1913]) (later taken up by Plessner, and now common currency in both the phenomenology and sociology of the body: see Wehrle 2019). They are ‘Leib’: the living shape of our sensual and sensate consciousness, the very substance of our subjective presence and mobile activity in the world. Otherwise put, it is the bodies and not the heads that should have been drawn first.

### The body as extended

One of the things that stands out in our data is the way that material spaces, their dimensions and objects, must be understood as *living spaces.* That is, spaces blended into, and acting as extensions of a kind of co-embodied system. Spaces then, that are imbued with intercorporeal and intersubjective meaning and that are part of the communicative field of the family home. This idea that the mind is embodied and that the embodied mind is extended and distributed through the body’s environment, has gained much traction in recent years (Clark 2011), tracking similar trends in sociology to take *things* seriously (Komter 2001). Behnke (2008) notes that living spaces are often places where numerous bodies extend, and interact with one another through these extensions, forming an ‘inter-kinesthetic field’. To understand the interacting and communicative body then, such as we find it in cases of self-injury, we have to understand this inter-kinesthetic field that embodied subjects create through their dwelling activity and the ‘sedimented traces’ of this activity (Meyer et al. 2017, p. xvii). While the inter-kinesthetic can be inferred from the intercorporeal and the intersubjective, as it has been here, it should perhaps be acknowledged as its own level or region of communicative activity in future research on self-injury.

### The body as relational

If the *Leib* or lived body is foundational to subjectivity, and it is something extended though a co-embodied system to create inter-kinesthetic fields out of shared spaces, then it is something that is not just shaped by biology but also by *action and interaction.* As Behnke puts it, ‘my body is something *I do*’, but crucially he adds,‘I do not do it alone’ (1997, p. 198, our emphasis). Perhaps ironically then, given how central the epistemology of the *Körper* (Foucault 2012 [1963])and the discourse of the *homo clausus* have been, it is *the body* that most clearly discloses that we are not wholly bounded individuals at all, beings onto whom relationships are ‘added’,^2^ but rather beings who can be individuals only because we are embedded within a coembodied matrix of social and worldly relationships. While it is though this matrix that individual subjects learn to distinguish between themselves and others, the shared infrastructure of intercorporeal, intersubjective and inter-kinesthetic communication remains active and so people never fully separate from one another (Kinsbourne and Jordan 2009). This is not to suggest however that the *personal* should be wholly effaced in favour of the *social*: rather, it is to draw out all the ways the personal and the social are mutually implicated, informed and entangled. As Zahavi explains:

[I]t is not possible simply to insert intersubjectivity [including the primary intersubjectivity of the intercorporeal] somewhere within an already established ontology; rather, the three regions, ‘self’, ‘others’ and ‘world’ belong together; they reciprocally illuminate one another, and can only be understood in their interconnection (2001, p. 166).

Nor is it to suggest that such a co-embodied system implies a sort of primal social harmony. As Alphonso Lingis notes, ‘[t]he cartography that maps out the distances and directions across which we identify and constrain one another maps out the ways that we [both] torment and gratify one another’ (1994, p. 54).

### The body as communicative

As the evidence of intercorporeal, intersubjective and inter-kinesthetic communication empirically sketched and theoretically described above demonstrates, our bodies are fundamentally *communicative bodies* (Frank 1991; O’Neill 1989). Describing what this means, O’Neill notes that the body is the ‘necessary organ’ of our social and discursive lives, ‘the bio-text upon which the principal social institutions inscribe themselves’ (1989, p. 3). So, we have the bodies that we have because of the communication of discourse and flesh; but equally, we have the institutions and discourses that we have for the same reason (ibid). However, as Frank makes clear,when the normative regulation of this feedback loop between discourse and body fails, the body does not simply become passive but rather speaks out with a code of its own making: ‘[t]he body continues to be formed among institutions and discourses, but *these are now media for its expression*. For the communicative body institutions and discourses now enable more than they constrain’ (1991, p. 80, our emphasis). If the discourses of the *homo clausus* and the sender–receiver communication paradigm are still present in the phenomenon of self-injury then, they are nonetheless transformed by the process of the communicative body ‘acting out’ in its estranged mode. They become, as Steggals argued, less a set regulative norms than the source material for a kind of transgressively hyperbolic bricolage. It is not that the truth of self-injuryis contained within these discourses then, nor can self-injury be understood when such discourses are taken for ontology. Rather, these discourses have become the productive and enabling media for the communicative body to ‘act out’ and speak up, to disclose and draw itself out beneath the talking head that has fallen silent.

## Conclusion

If self-injury has commonly suggested to us a *purely* private and secret phenomena, we must remember that the vernacular use of ‘private’ and ‘secret’ has never implied a hermetically sealed individualism.The phrase ‘private life’ tends to refer to people’s close and intimate relationships, while secrets are often shared amongst confidants without them being considered either inauthentic, or secrets no longer. The capacity and the desire to mask things or keep them secret is itself an accomplishment of social communicative capabilities because ‘secrecy constitutes a relational experience between people’ (van Manen and Levering 1996, p. 11). Smart has even suggested that we need to investigate the social history of secrets in the same way that Elias investigated the social history of manners, as part of the civilising process that forms *homo clausus* (Smart 2007, p. 110).

The disturbances and excitations of the communicative field of the family home that we have described here, demonstrate that self-injury cannot be thought of as something that is *either* individual *or* social, secret *or* communicated, private *or* shared. What we have found is that the personal is not something wholly contained within the boundaries of the body, but something that extends outward through other bodies, relationships and spaces. At the same time, the social is not something wholly exterior but rather something that saturates us, informing our most private thoughts, feelings and orientations. The personal and the social necessarily implicate one another. As such, while self-injury may be *experienced* as intensely personal, it is nonetheless best *conceptualised* as a richly communicative phenomenon: even if that communication is not an overt and fully deliberate interpersonal communication of the kind envisaged by the sender–receiver paradigm. A successful sociology of self-injury then, must include alongside any analysis of interpersonal communication, an appreciation of the intercorporeal, intersubjective, inter-kinesthetic. And this means that any account of the body in self-injury must understand this body as foundational to subjectivity, meaningfully extended through its environment, fundamentally relational, and deeply communicative. Indeed, self-injury offers a particularly powerful case study with which to think through a more complex model of communication and the role of the communicative body; something that ought to be of significant interest to the full range of sociologies of everyday life and embodiment.

## Footnotes

1 Our approach is one of sociological rather than philosophical phenomenology. As such our use of the term ‘intersubjective’ is empirical rather than eidetic.

2
Strathern (1992) regards this as a fundamental understanding of the person within English kinship.
